# Comparative mitogenomic and phylogenetic insights from four newly sequenced tick mitochondrial genomes

**DOI:** 10.3389/fvets.2025.1678349

**Published:** 2026-01-22

**Authors:** Shuang Liu, Rui Hou, Quanfu Zhang, Mingna Duan, Shaobo Tang, Weishuo Wang, Jiayao Chen, Jun Wu, Yujuan Shen, Wei Gu, Yi Sun, Xing Yang

**Affiliations:** 1 Integrated Laboratory of Pathogenic Biology, College of Preclinical Medicine, Dali University, Dali, China; 2 School of Health Management, Lanzhou College of Foreign Studies, Lanzhou, China; 3 Department of Gastroenterology, Clinical Medical College and the First Affiliated Hospital of Chengdu Medical College, Chengdu, Sichuan, China; 4 School of Public Health, Dali University, Dali, China; 5 Dali Bai Autonomous Prefecture People's Hospital Ophthalmology, Xiaguan, Dali, Yunnan, China; 6 National Key Laboratory of Intelligent Tracking and Forecasting for Infectious Diseases, Key Laboratory of Parasite and Vector Biology, Collaborating Centre for Tropical Diseases, National Institute of Parasitic Diseases at Chinese Center for Disease Control and Prevention, Chinese Center for Tropical Diseases Research, National Health Commission of the People's Republic of China, World Health Organization, Shanghai, China; 7 Department of Infection of the First Affiliated Hospital of Dali University, Yunnan Provincial Department of Education Key Laboratory of Infectious Diseases and the Clinical Medical Sub-Center of Infectious Diseases of Yunnan Province, Dali, China; 8 State Key Laboratory of Pathogen and Biosecurity, Academy of Military Medical Sciences, Beijing, China; 9 Yunnan Key Laboratory of Screening and Research on Anti-pathogenic Plant Resources from Western Yunnan, Dali, China

**Keywords:** characterization, tick, evolution, mitogenomes, phylogeny, genome annotation

## Abstract

Ticks, recognized as the second most significant vector of pathogens after mosquitoes, are of considerable interest in medical research. Although mitochondrial genomes are commonly employed in the phylogenetic studies of insects and arthropods, investigations into tick mitochondrial genomes are relatively scarce. The evolutionary and phylogenetic relationships at the family and genus levels remain unresolved. In this study, the mitochondrial genomes of *Haemaphysalis warburtoni, Ixodes crenulatus, Rhipicephalus bursa*, and *Rhipicephalus pumilio* were first sequenced and annotated based on the Illumina NovaSeq 6,000 platform, and compared with the mitochondrial genes of 150 other hard ticks. All examined tick mitochondrial genomes exhibit a notable AT bias, with A+T content ranging significantly from 72.28% to 81.06%. They also exhibit distinct codon usage patterns, with most codons ending in either A or U. A phylogenetic analysis, based on 13 protein-coding genes (PCGs), confirms that the Ixodidae family forms a monophyletic group. Based on the phylogenetic analysis of 13 protein-coding genes, the relationships of Ixodidae family as follows: *Ixodes* + (*Robertsicus* + ((*Bothriocroton* + *Archaeocroton* + *Cryptocroton* + *Haemaphysalis*) + (*Amblyomma* + (*Dermacentor* + (*Rhipicentor* + (*Hyalomma* + *Rhipicephalus*)))))). This study provides in-depth insights into tick mitochondrial genomes, offering important references for research on their systematics, evolution, and species identification, while also laying the groundwork for tick-borne disease control and public health risk assessment.

## Introduction

Ticks (Acari: Ixodida) are obligate hematophagous arthropods that are distributed throughout the world and parasitize almost every type of vertebrate except fish (1). As the second largest vector of pathogen transmission after mosquitoes, ticks have a serious impact on human and animal health (2). It causes reactions such as poisoning, irritation, sensitization and paralysis (which can be severe enough to lead to death) by parasitizing outside the body and biting and sucking blood. In addition, ticks can transmit many pathogens including rickettsia, spirochetes, viruses, bacteria and deadly protozoa, which can cause human disease such as Lyme disease (*Borrelia burgdorferi*), Rocky Mountain spotted fever (*Rickettsia rickettsii*), Mediterranean spotted fever (*Rickettsia conorii*), human granulocytic anaplasmosis (*Anaplasma phagocytophilum*), tularemia (*Francisella tularensis*), Colorado tick fever (Reoviridae, Orbivirus), and tick-borne encephalitis (Flaviviridae, Flavivirus). Additionally, in animals, they can lead to babesiosis (*Babesia* spp.), theileriosis (*Theileria* spp.), heartwater (*Ehrlichia ruminantium*), anaplasmosis (*Anaplasma* spp.), Lyme disease (*Borrelia burgdorferi*), and ehrlichiosis [*Ehrlichia* spp.; (3–7)]. All of these pathogens are detrimental to the health of humans, domestic animals, and wildlife. Factors such as global warming and increased human contact with nature have heightened the chances of tick bites and related diseases (8, 9). Furthermore, global expenditures on the management of ticks and the control and treatment of tick-borne diseases exceed $7 billion annually, imposing an additional heavy economic burden on the livestock industry (10, 11). Despite significant advances in the study of ticks and tick-borne pathogens, new tick-borne viruses have been discovered in recent years (12–14). Due to the important role that ticks play in veterinary medicine, medicine and veterinary medicine, species identification and taxonomic studies of ticks are of particular importance.

To date, ticks have been classified into 3 families, 18 genera and over 900 species according to traditional described and categorized (15). Based on the morphological description with a hard shield for the Ixodidae family and without a hard shield for the Argasidae family, the family Nuttalliellidae has its own unique characteristics and has only one species (*Nuttalliella namaqua*) recorded (4, 16). In recent years, scholars have discovered a new tick species (*Deinocroton draculi*) in Burmese amber ore that is about 99 million years old, a discovery that has allowed them to establish a new tick family-the Deinocroton family (17).

Initially, phylogenetic relationships among ticks were inferred based on their morphology, host types, and life cycles. The constructed “family tree” revealed two genera of soft ticks and five subfamilies with thirteen genera of hard ticks (18). However, it is unreliable to speculate on the phylogeny of ticks based on judgments such as tick “key morphological characters” or “host type” alone. Since the 1990s, molecular biology has been gradually applied to the study of ticks, and as a complementary method, molecular markers have been effective in making accurate identifications of parasites and clarifying the taxonomic status of some (19–22). However, most previous molecular studies have been limited to the use of relatively small datasets, such as 12S rRNA, 16S rRNA, cytochrome c oxidase subunit (*cox1*) (23–25). These data have helped solve some of the phylogenetic problems of ticks to some extent, but because these molecular markers are usually short and evolve rapidly, they are often not responsive to a deeper understanding of phylogenetic relationships.

Mitochondria are important organelles in eukaryotic cells. As semi-autonomous organelles, mitochondria have their own genetic material, known as the mitochondrial genome [mtDNA; (26)]. Over the past decade, the mitochondrial genome has been widely used in animal species identification, species reclassification, phylogenetic studies, and population genetics due to its structurally simplicity, strictly matrilineal inheritance, high degree of conservation, and relatively small molecular (27–31). The mitochondrial genome of ticks is structurally consistent with the number and sequence of metazoans, with a classic 37 genes (32) were the first to use complete mitochondrial genome sequences to study the phylogeny of hard tick lineages. Subsequent comparative analyses of mitochondrial genome sequences revealed that only by using the mitochondrial genome could the controversial phylogenetic relationships within soft tick lineages be effectively resolved (33). Subsequently, more scholars analyzed more mitochondrial genes of ticks, and combined with morphology, conducted more accurate inferences and analyzes on the phylogeny of families, subfamilies, genera and subgenus of *lxodes ticks* (31, 34–36). At the time of writing, the mitochondrial genome sequences of 184 tick species have been published in GenBank. The mitochondrial genome data provide reliable molecular for phylogeny, but there are a number of ticks for which mtDNA is still lacking, creating an important to refining the phylogenetic relationships between a wider range of tick lineages.

*Ixodes crenulatus* parasitizes companion animals such as cats and dogs, and can readily transmit tick-borne pathogens between humans and animals (37). Additionally, *Rhipicephalus bursa* transmits *Babesia ovis* (38), while *Rhipicephalus pumilio* serves as a vector for *Anaplasma ovis* (39). These three tick species pose threats to both human and animal health. However, current knowledge of their mitochondrial genomes, including that of the less-studied *Haemaphysalis warburtoni*, remains uncharacterized, with no relevant data reported in public databases. Therefore, elucidating the complete mitochondrial genomes of these tick species is of great importance for subsequent research on pathogen transmission mechanisms and tick evolution. This study reports the first complete mitochondrial genome sequences of four tick species: *Haemaphysalis warburtoni, Ixodes crenulatus, Rhipicephalus bursa*, and *Rhipicephalus pumilio*. Combined with previously published data from 150 hard tick species, a comprehensive phylogenetic analysis was conducted. Comparative genomic analyses were performed on newly sequenced and publicly available tick mitochondrial genomes to explore the diversity and evolutionary patterns within tick species. This research not only expands the molecular database for ticks but also provides foundational genetic markers for species identification, taxonomic classification of tick genera, and phylogenetic analysis.

## Materials and methods

All four newly sequenced specimens were from China, and detailed information is listed in Supplementary Table 1. The collected specimens were morphologically characteristics according to key morphological features and verified using *cox1* sequencing (40). The specimens were then preserved in 95% ethanol and stored at−80 °C until use. The collected specimens were divided into two copies, one for DNA extraction according to the instructions, and the other was stored as a voucher specimen in the Parasite Museum of Dali University (voucher numbers DLUP3010-DLUP3013). Total genomic DNA was extracted from individual ticks using the TIANamp Genomic DNA Kit according the manufacturer's instructions.

## Construction of the genomic library and sequencing

Total DNA from each specimen was extracted and normalized to a concentration of 0.3 ng/L for genomic library construction. Libraries were constructed using a whole genome shotgun (WGS) strategy on the Illumina NovaSeq 6,000 sequencing platform. These libraries, composed of 400 bp fragments, underwent paired-end sequencing. The sequencing output was transferred to a computer workstation, where data from each sample were counted and assessed individually. AdapterRemoval v2.0 was employed to eliminate splice-contaminated sequences (41), while FastQC software (https://www.bioinformatics.babraham.ac.uk/projects/fastqc) was used for data quality control. The average quality scores were 35.966 for *H. warburtoni*, 35.507 for *I. crenulatus*, 35.576 for *R. bursa*, and 35.780 for *R. pumilio*. Overlap clusters and scaffold sequences were generated using A5 miseq v20150522 (42) and SPAdes v3.9.0 (43), resulting in high-quality, assembled sequencing data. The average sequencing depth was 1060.21 X (*H. warburtoni*), 5938.68 X (*I. crenulatus*), 114.68 X (*R. bursa*) and 392.35 X (*R. pumilio*). Based on sequencing depth, sequences were extracted, and those with high depth were subjected to BLASTN analysis against the NCBI (nt) database to identify mitochondrial sequences from each assembly. The complete mitochondrial genome sequences and annotations for the four newly sequenced tick species have been deposited in the NCBI database under the following GenBank accession numbers: *H. warburtoni* (NC084204), *I. crenulatus* (OR872332), *R. bursa* (OR773535), and *R. pumilio* (NC084205).

## Annotation and bioinformatic analysis

Functional annotation of the obtained complete mitochondrial genome sequence using the MITOS web server (http://mitos.bioinf.uni-leipzig.de/index.py) (44). tRNAscan-SE Search Server v2.0 was used to predict the secondary structure of tRNAs (45). The NCBI BLAST online web page was used to search and compare sequence similarities, while protein-coding genes (PCGs) were analyzed and translated using an online open reading frame searcher. DNAStar V.5.0 software was used to calculate the AT and GC content of each gene, and the nucleotide skew values were analyzed using the formula AT-skew = (A – T)/(A + T) and GC-skew = (G – C)/(G + C) (46). The calculated values of AT-skew, GC-skew and AT% were counted and expressed in Origin Pro v9.0 using 3D scatter plots. To analyze the selection pressure, Ka and Ks values of 13 PCGs were calculated using DNASP V5.0 (47).

## Phylogenetic analysis

Phylogenetic relationships were constructed for 154 mitochondrial genomes of hard ticks (including the 4 sequenced in this study), with *Argas africolumbae* (NC019642), *Ornithodoros compactus* (NC067908), *Nuttalliella namaqua* (NC019663) and *Limulus polyphemus* (NC003057) as outgroups. Taxonomic information and GenBank data accession numbers for each species analyzed are listed in Supplementary Table 2. DAMBE v.7.2.102 was used to assess substitution saturation at each of the 13 mitochondrial PCG positions. Phylogenetic relationships between species were inferred using both maximum likelihood (ML) and Bayesian inference (BI) methods. The most appropriate model was inferred to be GTR+I+G based on ModelFinder, and the phylogenetic relationships of BI were constructed using MrBayes v3.2.6 (48) with four independent Markov chain Monte Carlo (MCMC) running simultaneously for 10,000,000 generations, with samples every 1,000 generations. The first 25% of the flutter trees were aged and the remaining trees were used to compute Bayesian posterior probabilities. Phylogenetic relationships for ML are constructed using IQ-Tree (49), and 1,000 bootstrapped replicates are selected to evaluate the reliability of branch nodes (50). Finally, the phylogenetic tree was displayed using Figtree v.1.42.

## Results

### Mitogenome nucleotide composition and genome organization

**Figure 1:**
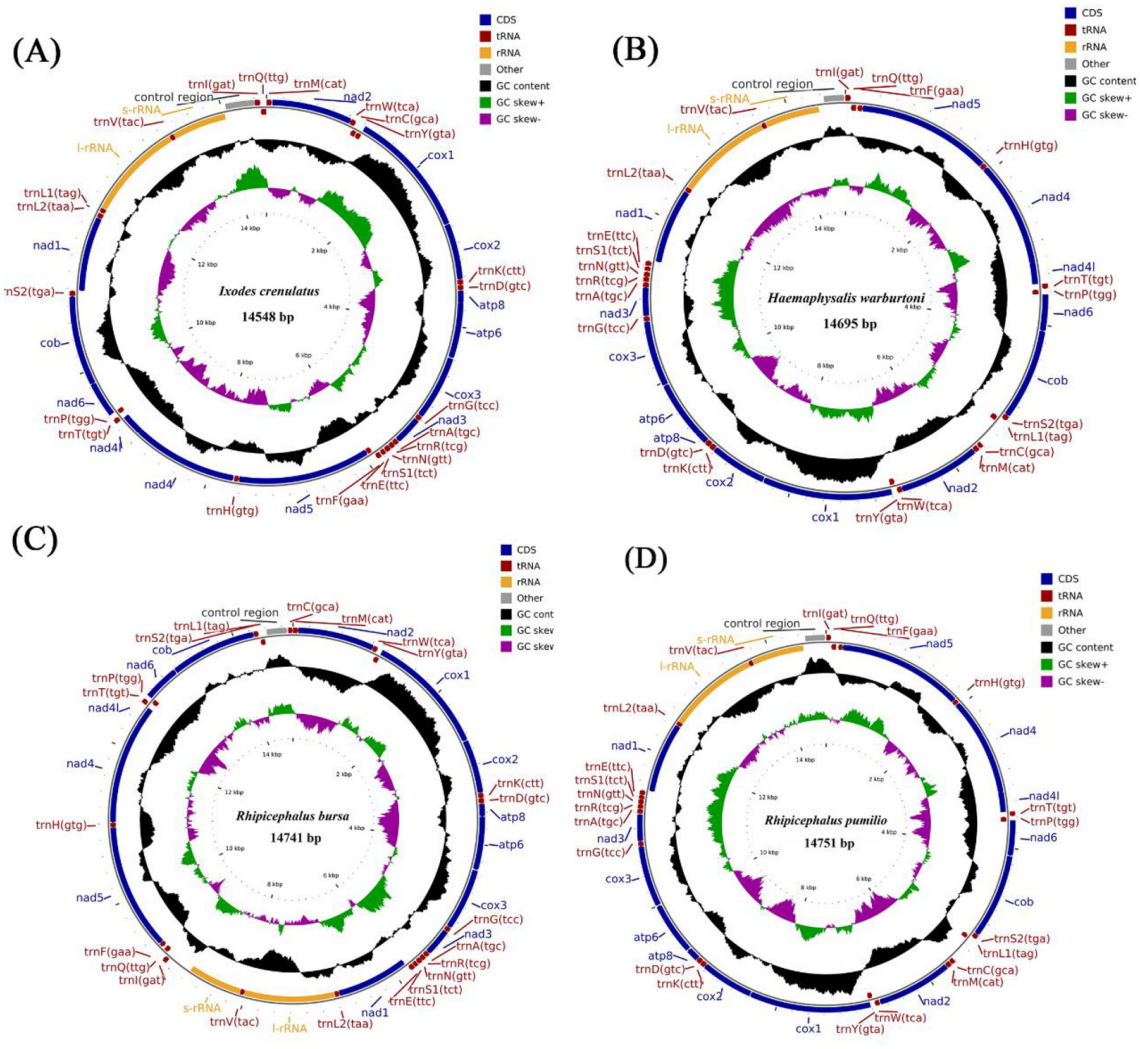
Mitochondrial genome structure of *Ixodes crenulatus* **(A)**, *Haemaphysalis warburtoni* **(B)**, *Rhipicephalus bursa* **(C)**, *Rhipicephalus pumilio* **(D)**. With genes located on the J (forward) strand are displayed on the outer side, while the N (reverse) strand genes are indicated in the inner. The colors of gene names represent control regions (gray), tRNA genes (red), PCGs (blue) and rRNA genes (yellow), respectively.

**Figure 2:**
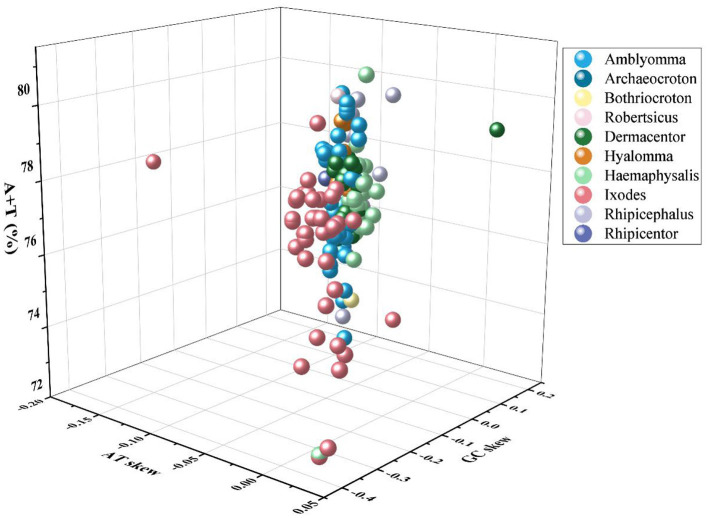
Three-dimensional scatter plot displaying AT-skew, GC-skew, and AT% composition across 154 mitochondrial genomes of hard ticks, with different colors representing the various genera of the mtgenomes.

This study is the first to sequence the mitochondrial genomes of *H. warburtoni, I. crenulatus, R. bursa*, and *R. Pumilio*, which adds to the diversity of mitogenomes reported, especially for *Ixodes, Haemaphysalis* and *Rhipicephalus* (Figure 1 and Supplementary Table 3). The mitochondrial genomes of all 154 tick species studied contain the typical 37 genes, including 13 protein-coding genes (PCGs), 2 ribosomal RNA genes (16S rRNA and 12S rRNA), and 22 transfer RNA genes (tRNAs), as well as a varying number of D-loops. Most of these genes are located in the positive strand of the mitochondrial genome, while a few are located in the negative strand. The mitochondrial complete genome sequence length varies among Ixodidae family ticks, ranging from 14,463 bp (*Ixodes loricatus*) to 15,307 bp (*Dermacentor sp*.), with an average length of 14,778 bp. In hard ticks, the A+T content shows significant variation, ranging from 72.28% (*Ixodes (Pholeoixodes)* sp.) to 81.06% (*Haemaphysalis danieli*), with an average of 77.72%. The average AT-skew and GC-skew are −0.017 and −0.171, respectively (Figure 2 and Supplementary Table 5).

### Protein-coding genes

In hard ticks (Ixodidae), the length of the mitochondrial complete protein-coding genome ranges from 10,639 bp (*Amblyomma marmoreum*) to 10,910 bp (*Bothriocroton concolor*), with an average length of 10,832 bp. The A+T content varies between 70.78% (*Ixodes (Pholeoixodes) sp*.) and 81.20% (*Haemaphysalis danieli*), with an average A+T content of 77.40%. The AT-skew is predominantly negative, and the GC-skew also mostly shows negative values.

**Figure 3:**
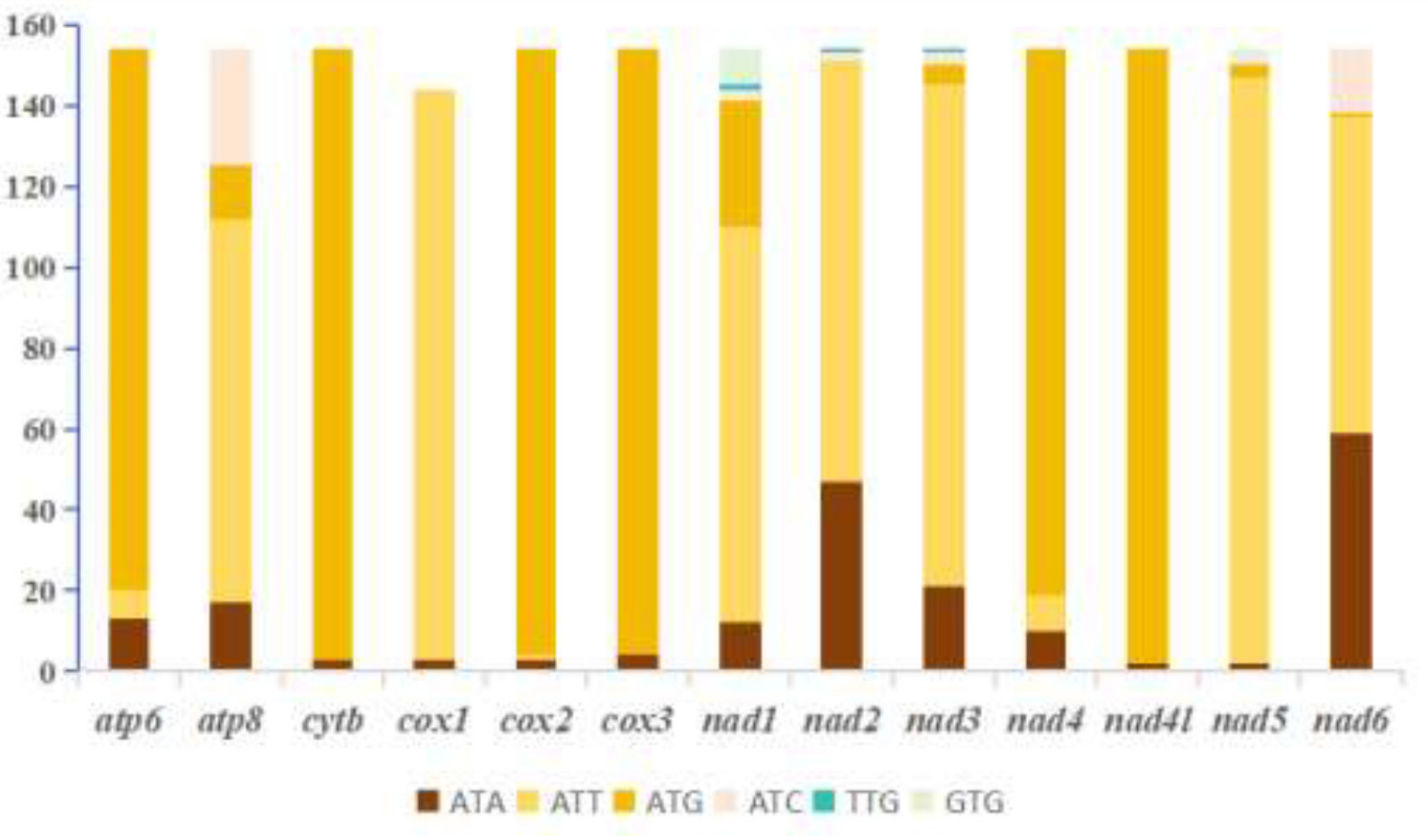
Analysis of start codon usage in the 13 protein-coding genes from 154 Ixodidae (hard tick) species.

**Figure 4:**
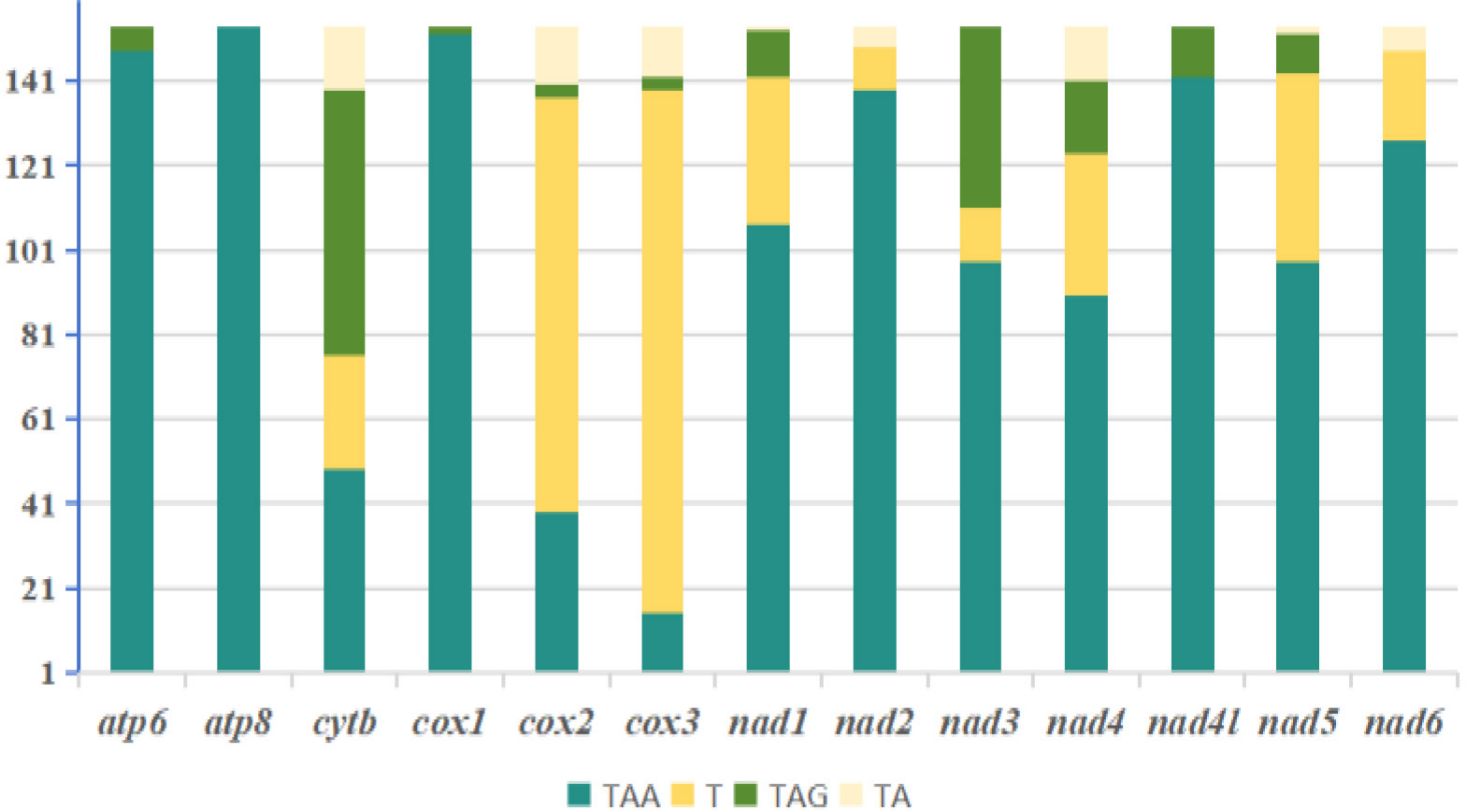
Analysis of stop codon usage in the 13 protein-coding genes from 154 Ixodidae (hard tick) species.

The initiation codon usage in Ixodidae species shows similarity at the species level. Nearly all species use ATN as the initiation codon, with ATT and ATG being more common. ATA is used more frequently in a few species, while ATC is the least used. Among the 13 protein-coding genes (PCGs), *atp6, cox2, cox3, cytb, nad4*, and *nad4l* preferentially use ATG as the initiation codon. Notably, the NADH dehydrogenase subunit genes (*nad1, nad2, nad3*, and *nad5*) more frequently use ATT as the initiation codon, A few genes, namely *nad5, nad3, nad2*, and *nad1*, employ the relatively rare initiation codon GTG (Figure 3). In all species, TAA is the most common termination codon for mitochondrial protein-coding genes, while TA is used less frequently. Among the 13 PCGs, *atp6, atp8, cox1, nad2, nad4l*, and *nad6* very frequently use TAA as the termination codon. Particularly, *cox2* and *cox3* tend to use the incomplete termination codons T and TA (Figure 4).

**Figure 5:**
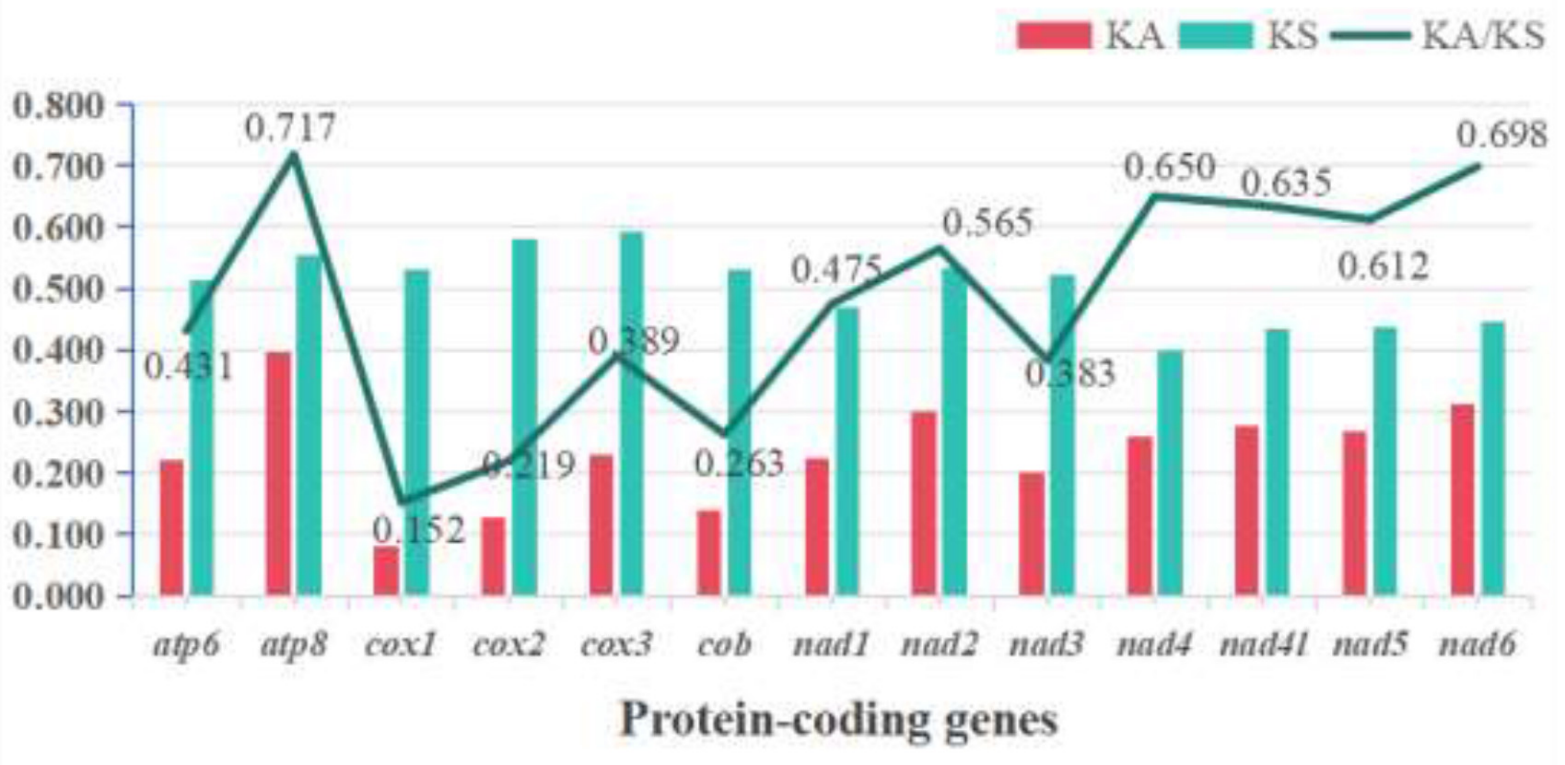
The evolutionary rates of the 13 protein-coding genes (PCGs) across the 154 Ixodidae mitochondrial genomes were assessed using the Ka/Ks ratio, with values interpreted as follows: Ka/Ks = 1 signifies neutral evolution; Ka/Ks < 1, purifying selection; and Ka/Ks > 1, positive selection.

**Figure 6:**
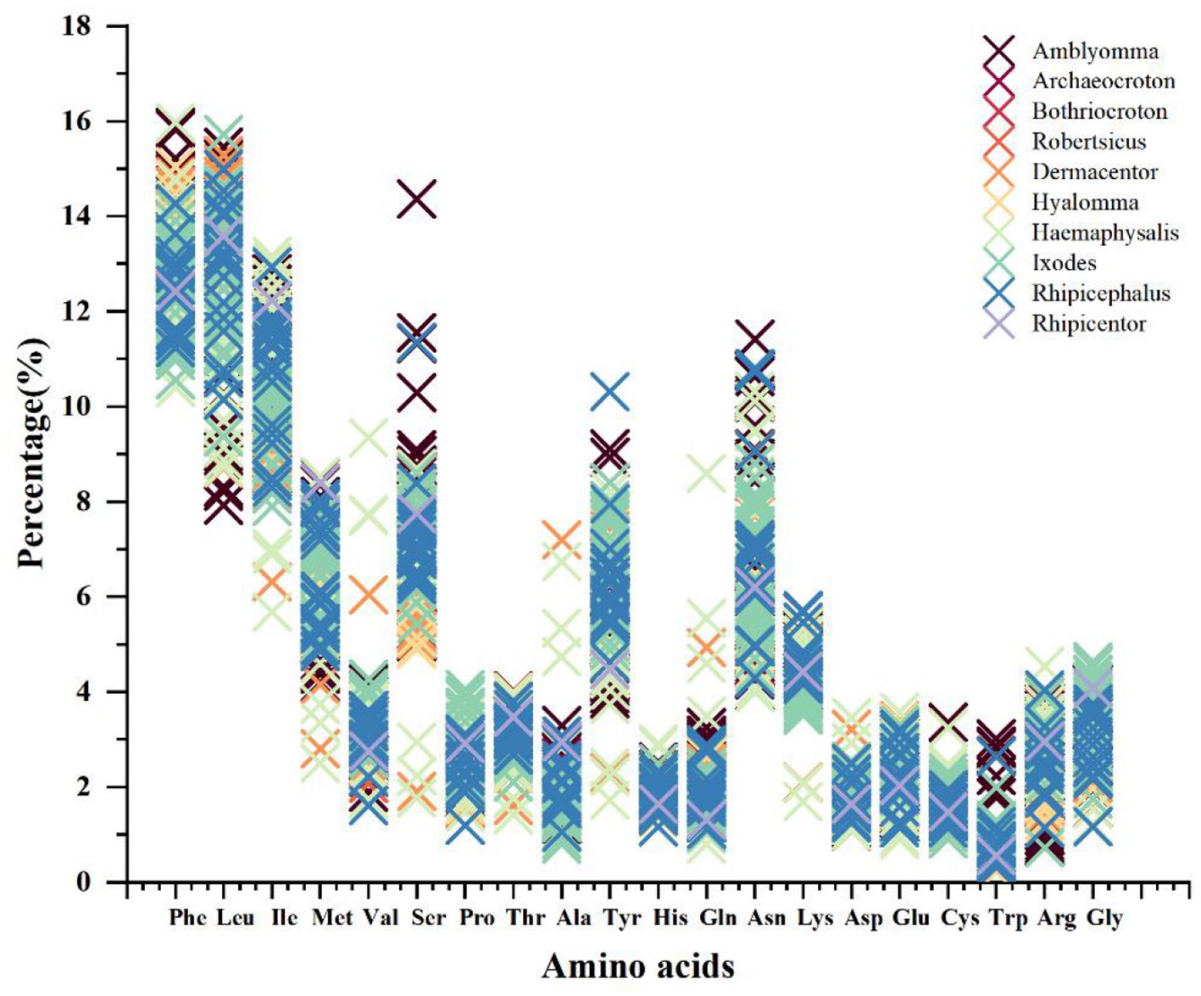
Frequency percentage of each of the 20 coded amino acids in 154 mitochondrial genome sequences of in the family Ixodidae.

By calculating the synonymous (Ks) and non-synonymous (Ka) substitution rates for the mitochondrial protein-coding genes across the Ixodidae family, we estimated their evolutionary rates. The results indicated that *atp8* exhibits the highest evolutionary rate, whereas *cox1* shows the lowest. Although all Ka/Ks values were less than 1, the other PCGs exhibited considerable variation in their evolutionary rates (Figure 5). The amino acid usage frequency was calculated for the 13 PCGs. The results indicated a similarity in amino acid usage across the 154 hard tick species. The most prevalent amino acids were leucine (Leu), phenylalanine (Phe), and isoleucine (Ile), while tryptophan (Trp) was the least frequent (Figure 6).

**Figure 7:**
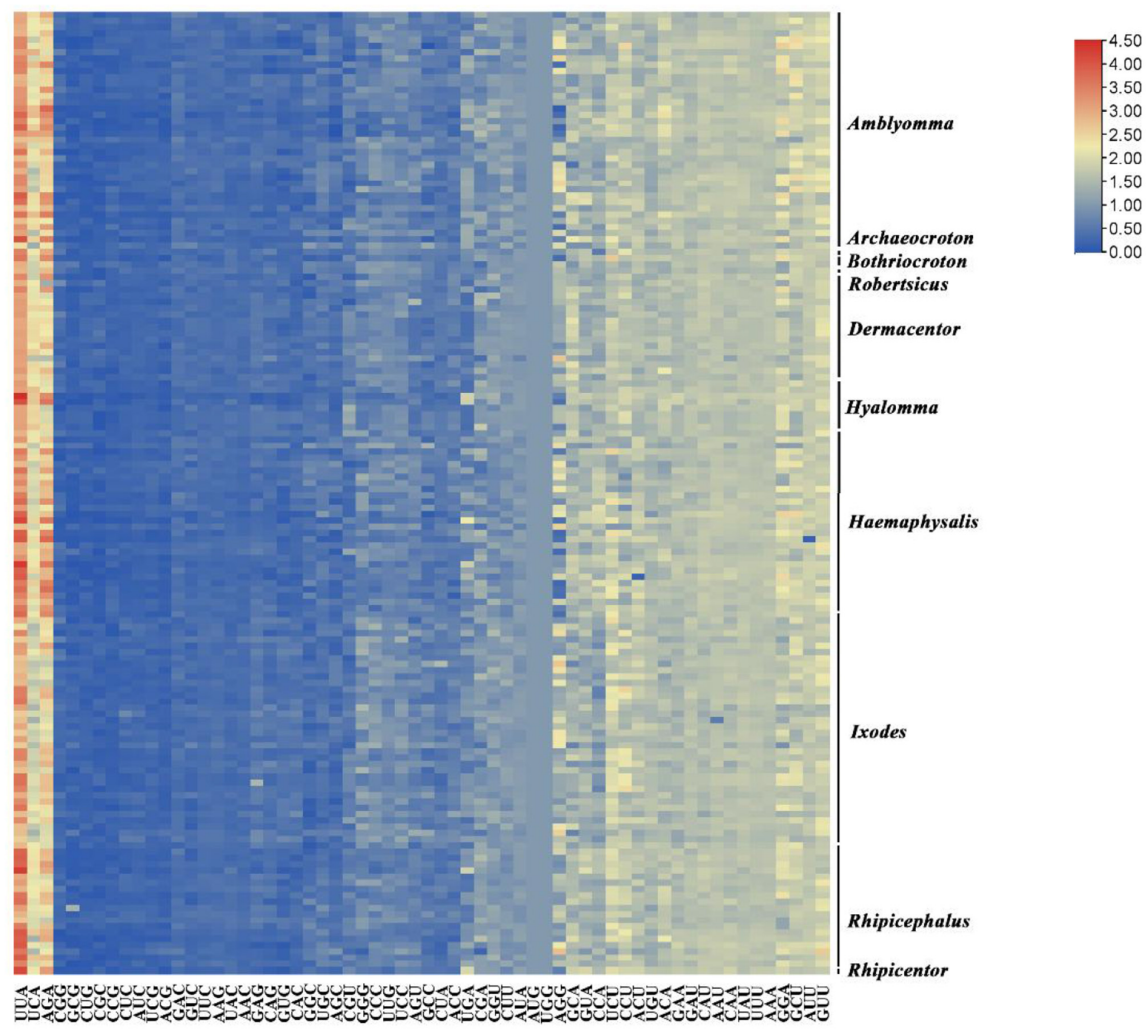
Comparative analysis of synonymous codon usage (RSCU) preferences and conservation in the mitochondrial protein-coding genes of 154 Ixodidae tick species.

Relative Synonymous Codon Usage (RSCU) can intuitively reflect codon usage bias. As shown in Figure 7, codon clustering based on RSCU values (represented by color gradients) reveals the following characteristics: light yellow indicates high-frequency usage (RSCU > 1), orange represents relatively high-frequency usage (RSCU > 2), and blue denotes low-frequency usage (RSCU < 1). The codons AUG and UGG show consistent usage frequencies across species, both with an RSCU value of 1. The most frequently used codon is UUA, while the least frequently used is CCG. Notably, the high-frequency codons UUA, UCA, and AGA (all ending with A at the third nucleotide position), along with most preferred codons in the right cluster, predominantly terminate with A or U. This demonstrates a significant A or U-ending preference for optimal codons in hard ticks (Ixodidae; Figure 7 and Supplementary Table 4).

### D-loop, transfer and ribosomal RNA genes

**Figure 8:**
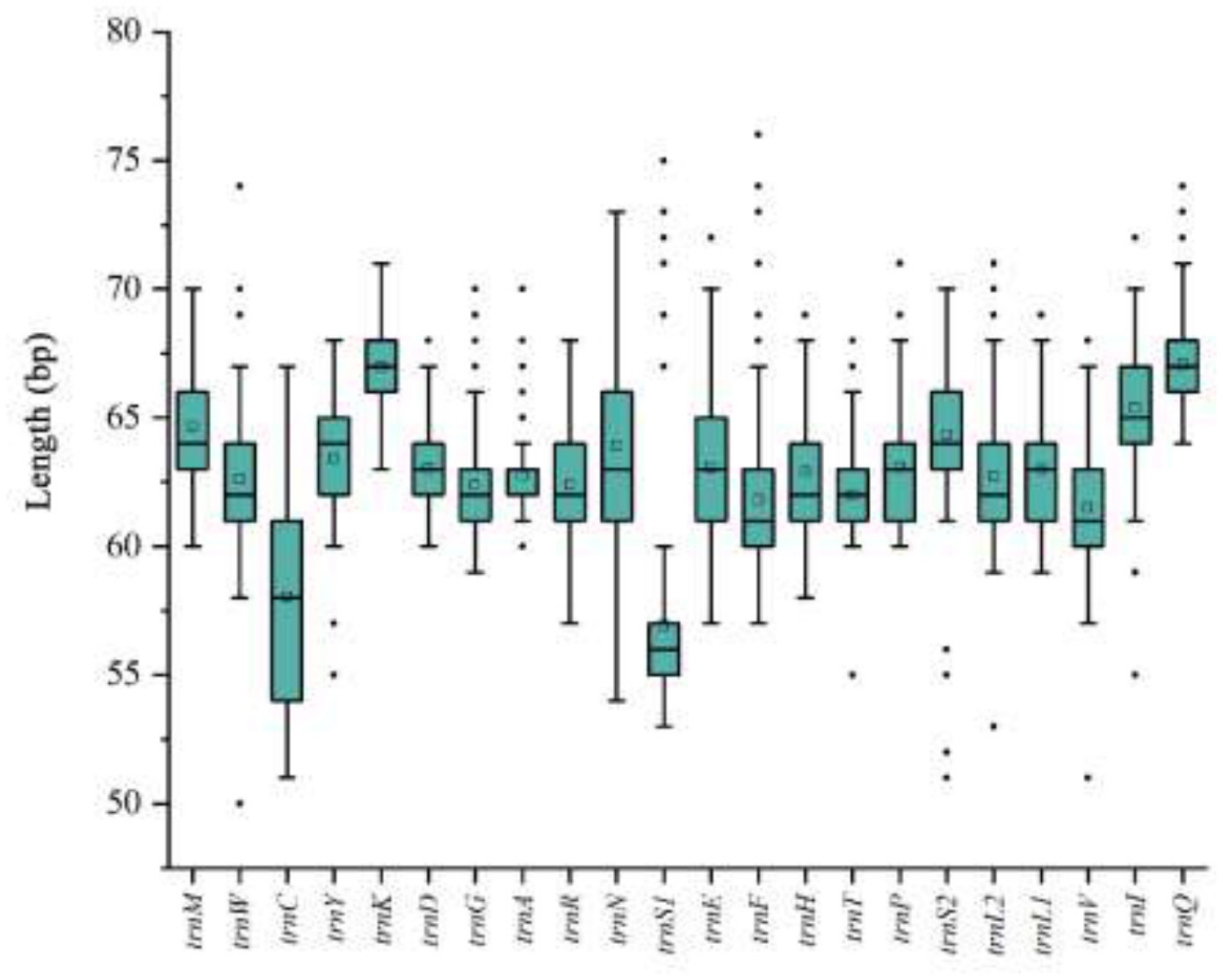
Composition of mitochondrial tRNA gene lengths across 154 hard tick species.

**Figure 9:**
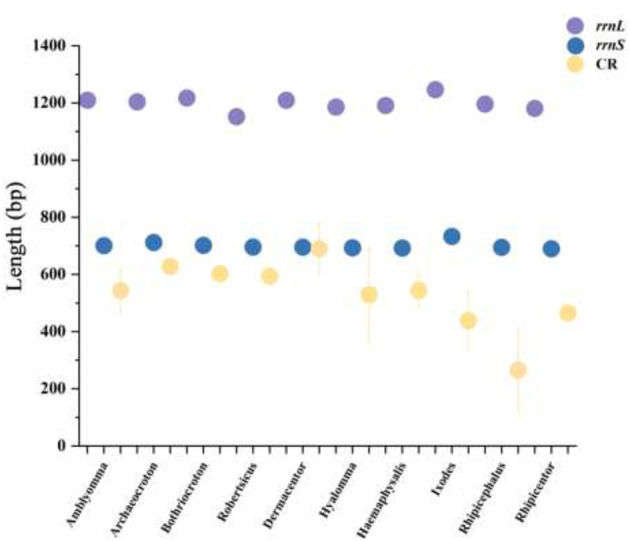
Composition of mitochondrial rRNA genes and control region (CR) lengths in 154 hard tick species.

The mitochondrial tRNA genes of hard ticks (Ixodidae) range in length from 50 to 76 bp (Figure 8). Most tRNA genes exhibit a complete cloverleaf secondary structure, with the presence of non-canonical base pairs (G-U, U-U, and U-G mismatches) that contribute to structural stability. The mitochondrial genome of hard ticks also includes two rRNA genes (16S rRNA and 12S rRNA). The 16S rRNA gene exhibits a length range of 982–1,381 bp and the 12S rRNA gene ranges from 649 to 1,201 bp in length (Figure 9). The mitochondrial genome typically contains 1–2 non-coding regions (NCRs), though some tick species possess 3 or more NCRs. The total length of these NCRs varies considerably from 210 to 1,412 bp, with the number of NCRs differing among genera without apparent regularity. Notably, in *Amblyomma calabyi*, an exceptionally short 1 bp intergenic spacer was observed between *trnL*_1_ and *trnC* genes, which may potentially represent a mitochondrial genome annotation artifact. It is worth noting that a unique common sequence marker, termed “Tick-box”, has been identified in the mitochondrial genomes of ticks. However, this marker was not found in the mitochondrial genomes of the 4 tick species analyzed in the present study.

### Mitogenomic phylogenetic analyses

**Figure 10:**
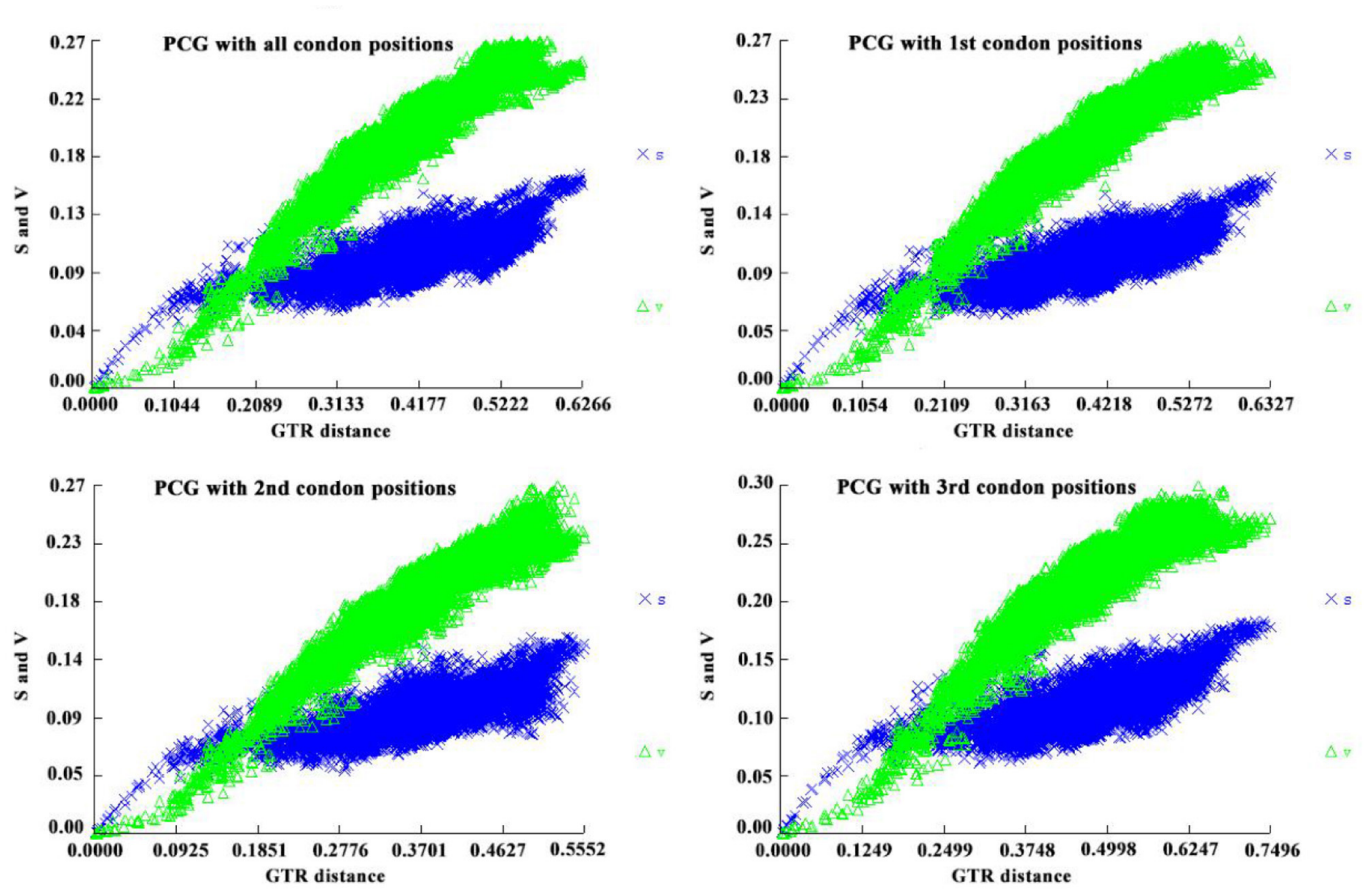
Substitution saturation test of different coding sites in the 13 protein-coding genes (PCGs) of 154 tick mitogenomes. In the figure, s and v represent transition distance and transversion distance, and general time reversible (GTR) distance represents the use of the GTR model to calculate the total genetic distance. **(A)** PCG with all codon positions; **(B)** PCG with 1st codon position; **(C)** PCG with 2nd codon position; **(D)** PCG with 3rd codon position.

None of the nucleotide databases were saturated, suggesting the suitability of constructing phylogenetic trees as a means of inferring relationships within ticks (Figure 10). We then constructed a phylogenetic tree using published data for 150 species available in NCBI to infer tick internal relationships using both ML and BI methods, and ultimately found that the use of the same sequence matrix yielded two highly similar topologies that differed slightly at the genus level.

**Figure 11:**
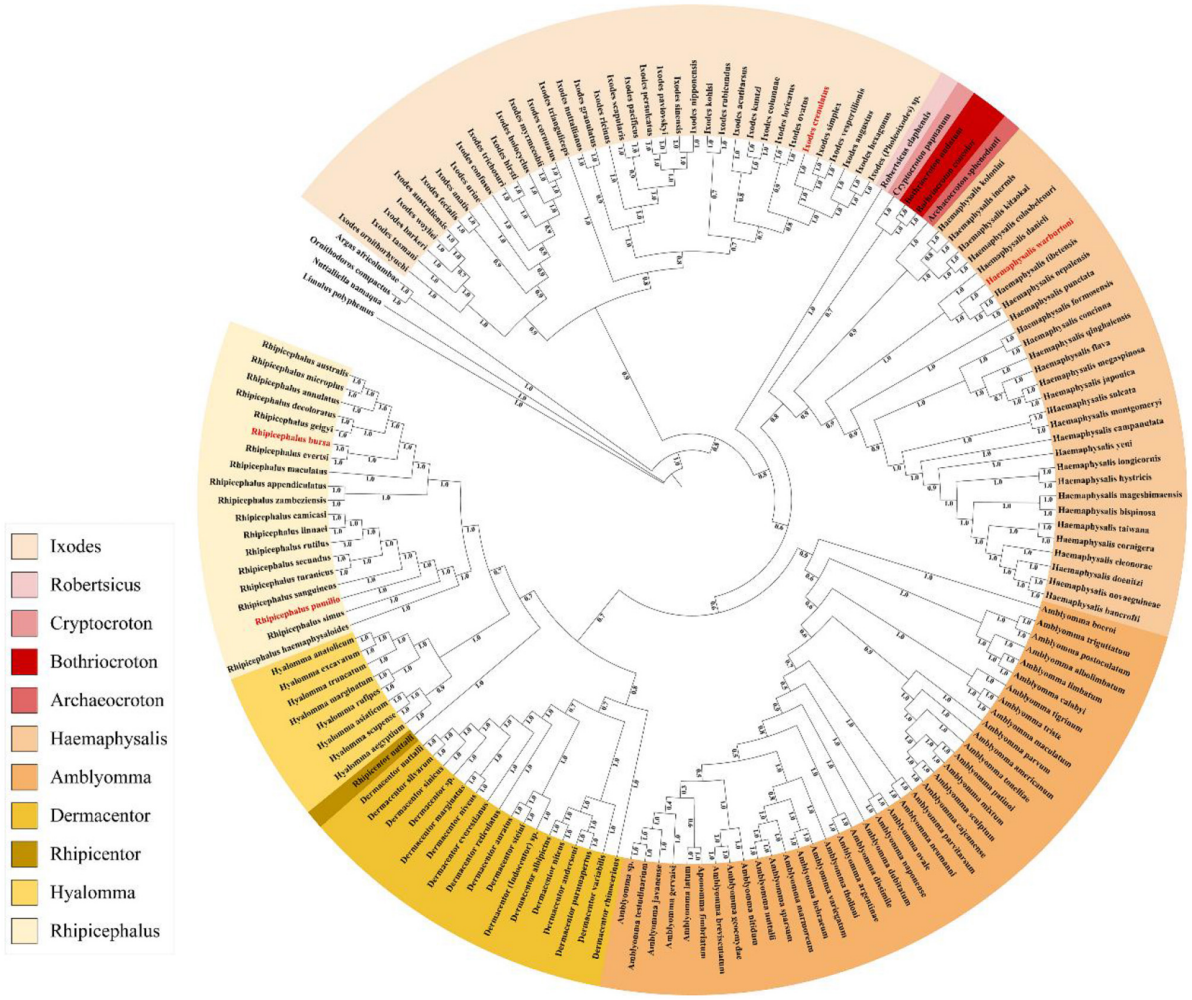
Mt-genome based phylogenetic relationships of 154 hard tick species. They are constructed based on 13 protein-coding genes dataset using Bayesian inference methods. The different colors of species name blocks represent the different genus. Red font indicates the 4 newly sequenced mt-genomes of hard ticks in this study. Species and NCBI accession numbers for mt-genomes used in the phylogenetic analysis are provided in Supplementary Table 2.

Within the hard tick family, both the ML and BI tree topologies exhibit the same two lineages: the Prostriata and Metastriata. Prostriata consists of only the hard tick genera and is the basis of all hard tick genera. Metastriata include *Robertsicus, Bothriocroton, Archaeocroton, Haemaphysalis, Dermacento*r, *Amblyomma, Rhipicentor, Hyalomma*, and *Rhipicephalus*. Most genera are monophyletic, with the (*Archaeocroton* + *Bothriocroton* + *Haemaphysalis*) forming a sister group relationship with the (*Amblyomma* + (*Dermacentor* + (*Rhipicentor* + *Rhipicephalus*))) branch. The relationship between *Bothriocroton, Archaeocroton*, and *Haemaphysalis* is not yet clear (Figure 11 and Supplementary Figure 1).

The phylogenetic analysis revealed several strongly supported relationships. *I. simplex* and *I. vespertilionis* formed a highly supported clade (100% bootstrap support, 1.00 posterior probability), which grouped with *I. crenulatus* (100% bootstrap support, 1.00 posterior probability); *R. bursa* is sister to *R. evertsi* (100% bootstrap support, 1.00 posterior probability); while *R. pumilio* was most closely related to *R. sanguineus* (77.72% bootstrap support, 1.00 posterior probability); *H. tibetensis* was sister to *H. nepalensis* (100% bootstrap support, 1.00 posterior probability), and this clade further grouped with *H. warburtoni* (100% bootstrap support, 1.00 posterior probability).

## Discussion

This study reports the first sequencing and annotation of the mitochondrial genomes of *Haemaphysalis warburtoni, Ixodes crenulatus, Rhipicephalus bursa*, and *Rhipicephalus pumilio*, integrating these data with 150 publicly available hard tick mitogenomes for a comprehensive systematic analysis. The mitochondrial genomes of the 154 tick species examined in this study varied in length from 14,463 bp to 15,307 bp. All tick genomes contained 37 genes. Length variations, primarily due to D-loops, spacer regions, and overlapping regions, were consistent with previous reports on ticks (51, 52). The nucleotides of all species studied showed a pronounced AT content preference, with high levels of A+T, particularly in postnatal animals, where A+T content typically exceeds G+C content. Additionally, soft ticks exhibited significantly higher A+T content than hard ticks, correlating with variations in their habitats, life strategies, and survival environments (53–55). Base skewing is mainly attributed to asymmetric base mutations during replication and transcription, as well as to selective pressures. Most hard ticks exhibit a negative AT-skew, a notable exception to the general metazoan pattern of a positive AT-skew and a negative GC-skew in their mitochondrial genomes. Similar phenomena have also been observed in other animal groups such as Araneae and Lepidoptera (56, 57).

In the 13 protein-coding genes (PCGs) of ticks, the start codons predominantly conform to the classical ATN format, whereas the stop codons are primarily TAA. Additionally, the presence of atypical initiation codons TTG and GTG was identified. These codons are corrected during mRNA editing, thus not affecting normal protein translation (58). In some protein genomes, incomplete stop codons “T” and “TA” have been noted. These incomplete stop codons are converted to TAA through post-transcriptional polyadenylation (59). The Ka/Ks ratios of PCGs reflect the selective pressures on genes. For ticks, the Ka/Ks ratios for individual protein-coding genes are below 1, indicating that ticks have primarily undergone purifying selection during their evolution (60). The *cox1* gene exhibits the lowest Ka/Ks ratio, highlighting its conservation and the strong functional constraints it has faced. Conversely, the *atp8* gene displays the highest Ka/Ks ratio, suggesting it has the greatest variation and has experienced relatively weak selective pressure.

Most tRNA genes in tick mitochondrial genomes possess the typical cloverleaf structure, including four main structural regions: the amino acid acceptor arm (AA), the TΨC arm (T), the anticodon arm (AC), and the dihydrouridine arm (DHU) (61). The absence of the DHU arm in *trnS1* is common among ticks, a trait shared with most insects and metazoan mitochondrial genomes (62, 63). Similarly, *trnC* often lacks the D-arm, a feature believed to be an ancestral trait within the Mesostigmata (64). In insects, mitochondrial non-coding region transcription termination is facilitated by the binding of transcription factors to specific sites (65). In tick mitochondrial genomes, the size, number, and position of non-coding regions (NCRs) vary, with the most frequent location being between the *rrnS* and *trnI* genes. Typically, the length of NCRs within a single genus varies little, generally containing only 1 or 2 NCRs. However, in the genus *Dermacentor*, multiple non-coding regions have been identified, which may contribute to gene rearrangement processes and help protect gene function (55). A distinct marker sequence known as the “Tick-box” motif has been identified in the non-coding regions of tick mitochondrial genomes, likely arising from gene degeneration during evolution (66). This “Tick-box” motif, particularly when directionally repeated at the 3′ ends of the *nad1* gene and 16S rRNA gene in *H. inermis* and other species, may have facilitated transposition events, leading to the formation of partial gene copies (31).

The results of this study, which utilize the mitochondrial genome to infer phylogenetic relationships among genera within the Ixodidae family, are robust and align with prior morphological and molecular findings. The Ixodidae family is confirmed as a monophyletic group, with each genus forming well-defined branches (51). Specifically, the *Ixodes* genus is monophyletic, with Australasian *Ixodes* and non-Australasian *Ixodes* forming distinct clusters. However, the precise number of extant species within the Australasian *Ixodes* remains uncertain, as most hard ticks have not been thoroughly studied. In accordance with earlier research, a phylogenetic tree constructed from 13 protein-coding genes places the Ixodes genus at the basal position within the *Ixodidae* (29). *I. crenulatus* is identified as forming a sister group with *I. simplex* and *I. Vespertilionis* (100% BS, 1.00 PP). Previous research indicated that the Haemaphysalinae subfamily is closely related to the Hyalomminae and Rhipicephalinae subfamilies (18), based on morphological traits such as leg structure and palpal spurs. Current analyses based on PCGs further substantiate the phylogenetic placement of the *Ixodes* genus. Both the Maximum Likelihood and Bayesian Inference trees confirm that the *Ixodes* genus forms a monophyletic group and is sister to *A. sphenodonti, R. elaphensis, B. undatum*, and *B. consolor*, upporting current conclusions (29, 51). *H. warburtoni* is sister to *H. nepalensis* and *H. tibetensis* (100% BS, 1.00 PP), the genus Haemaphysalis may not be monophyletic. All *Amblyomma* species form a monophyletic group. *Amblyomma* species collectively form a monophyletic group. The Rhipicephalinae subfamily, including *Hyalomma* and *Rhipicephalus*, forms a monophyletic clade with *Dermacentor* and *Rhipicentor* (67, 68). *R. bursa* is sister to *R. Evertsi* (100% BS, 1.00 PP); while *Rh. Pumilio* was most closely related to *R. sanguineus* (77.72% BS, 1.00 PP), with all species of *Rhipicephalus* also forming a monophyletic group (69).

Ticks are a group of harmful pests that pose a significant threat to the health of both humans and animals. Currently, biological and chemical methods are widely used for tick control (70, 71); however, accurately identifying their species and clarifying their taxonomic status remain fundamental to effective prevention and control efforts. In this study, the mitochondrial genomes of *H. warburtoni, I. crenulatus, R. bursa*, and *R. pumilio* were sequenced for the first time, providing valuable genetic markers for species identification, population genetics, and molecular epidemiology of ticks. Previous mitochondrial genomic studies have supported the monophyly of the families Ixodidae, Argasidae, and Nuttalliellidae. The results of this study also support the monophyly of these three families. However, the phylogenetic relationship between the Nuttalliellidae family and other families remains unclear. Furthermore, more evidence is needed to verify whether the genera within the Ixodidae and Argasidae families form monophyletic or paraphyletic groups (31, 72). In recent years, mitochondrial genomes have been widely used in the systematic taxonomy and evolutionary studies of ticks due to their maternal inheritance and relatively conserved sequences. However, their high nucleotide substitution rate may lead to the accumulation of base substitutions and saturation of homologous sequences, making it difficult to accurately resolve deeper phylogenetic relationships, such as those at the subfamily level (51). Therefore, future research integrating mitochondrial genome data with nuclear genome data is expected to provide clearer insights into the phylogenetic relationships of ticks. Therefore, in future research, the integrated analysis of both mitochondrial and nuclear genomic data is expected to more clearly reveal the evolutionary relationships within tick phylogeny.

## Conclusion

This study is the first to sequence the mitochondrial genomes of *H. warburtoni, I. crenulatus, R. bursa*, and *R. Pumilio*, these data provide a genomic foundation for future molecular epidemiology, species delimitation, and evolutionary studies in ticks, particularly within the Ixodidae. With the widespread application of molecular data, mitochondrial genomes will effectively compensate for the limitations of traditional morphological identification and play important roles in areas such as accurate species identification and resolution of phylogenetic relationships.

## Data Availability

The datasets presented in this study can be found in online repositories. The names of the repository/repositories and accession number(s) can be found in the article/Supplementary material.
