# Supplementary material for: Comparative mitogenomic and phylogenetic insights from four newly sequenced tick mitochondrial genomes
Source: Front Vet Sci. 2026 Jan 22;12:1678349. doi: 10.3389/fvets.2025.1678349 (PMC12872541; doi:10.3389/fvets.2025.1678349)
Supplement: Supplementary file 2 [file Table_2.docx]

| Family | Genus | Species | GenBank accession |
| --- | --- | --- | --- |
| Ixodidae | *Amblyomma* | *Amblyomma tholloni* | NC_067901 |
|  | *Amblyomma* | *Amblyomma mixtum* | OP901703 |
|  | *Amblyomma* | *Amblyomma maculatum* | MW719251 |
|  | *Amblyomma* | *Amblyomma hebraeum* | NC_067897 |
|  | *Amblyomma* | *Amblyomma sparsum* | NC_067860 |
|  | *Amblyomma* | *Amblyomma latum* | OL741735 |
|  | *Amblyomma* | *Amblyomma gervaisi* | OL741734 |
|  | *Amblyomma* | *Amblyomma testudinarium* | MT029329 |
|  | *Amblyomma* | *Amblyomma ovale* | NC_050255 |
|  | *Amblyomma* | *Amblyomma javanense* | NC_043872 |
|  | *Amblyomma* | *Amblyomma sculptum* | NC_032369 |
|  | *Amblyomma* | *Amblyomma cajennense* | NC_020333 |
|  | *Amblyomma* | *Amblyomma americanum* | NC_027609 |
|  | *Amblyomma* | *Amblyomma triguttatum* | NC_005963 |
|  | *Amblyomma* | *Amblyomma parvum* | OR899812 |
|  | *Amblyomma* | *Amblyomma neumanni* | OR899811 |
|  | *Amblyomma* | *Amblyomma parvitarsum* | OR899810 |
|  | *Amblyomma* | *Amblyomma tigrinum* | OR899809 |
|  | *Amblyomma* | *Amblyomma dubitatum* | OR899808 |
|  | *Amblyomma* | *Amblyomma naponense* | OR899807 |
|  | *Amblyomma* | *Amblyomma boeroi* | OR899806 |
|  | *Amblyomma* | *Amblyomma argentinae* | OR899805 |
|  | *Amblyomma* | *Amblyomma dissimile* | OR899804 |
|  | *Amblyomma* | *Amblyomma patinoi* | NC_072689 |
|  | *Amblyomma* | *Amblyomma tonelliae* | NC_072690 |
|  | *Amblyomma* | *Amblyomma geoemydae* | MK814531 |
|  | *Amblyomma* | *Amblyomma nuttalli* | OL741736 |
|  | *Amblyomma* | *Amblyomma sp.* | OM368313 |
|  | *Amblyomma* | *Amblyomma marmoreum* | KY457516 |
|  | *Amblyomma* | *Amblyomma limbatum* | OR416215 |
|  | *Amblyomma* | *Amblyomma albolimbatum* | OR350524 |
|  | *Amblyomma* | *Amblyomma variegatum* | LC834208 |
|  | *Amblyomma* | *Amblyomma triste* | NC_088502 |
|  | *Amblyomma* | *Amblyomma papuanum* | NC_087879 |
|  | *Amblyomma* | *Amblyomma postoculatum* | NC_087876 |
|  | *Amblyomma* | *Amblyomma nitidum* | NC_087875 |
|  | *Amblyomma* | *Amblyomma calabyi* | NC_087874 |
|  | *Amblyomma* | *Amblyomma breviscutatum* | OR416214 |
|  | *Amblyomma* | *Aponomma fimbriatum* | JN863730 |
|  | *Archaeocroton* | *Archaeocroton sphenodonti* | NC_017745 |
|  | *Bothriocroton* | *Bothriocroton undatum* | NC_017757 |
|  | *Bothriocroton* | *Bothriocroton concolor* | NC_017756 |
|  | *Robertsicus* | *Robertsicus elaphensis* | NC_017758 |
|  | *Dermacentor* | *Dermacentor albipictus* | NC_067093 |
|  | *Dermacentor* | *Dermacentor reticulatus* | OM867334 |
|  | *Dermacentor* | *Dermacentor variabilis* | NC_061217 |
|  | *Dermacentor* | *Dermacentor andersoni* | NC_061057 |
|  | *Dermacentor* | *Dermacentor auratus* | NC_059724 |
|  | *Dermacentor* | *Dermacentor everestianus* | NC_042764 |
|  | *Dermacentor* | *Dermacentor nuttalli* | NC_028528 |
|  | *Dermacentor* | *Dermacentor silvarum* | KP258209 |
|  | *Dermacentor* | *Dermacentor sinicus* | NC_062165 |
|  | *Dermacentor* | *Dermacentor niveus* | NC_062070 |
|  | *Dermacentor* | *Dermacentor steini* | NC_062068 |
|  | *Dermacentor* | *Dermacentor marginatus* | NC_062069 |
|  | *Dermacentor* | *Dermacentor nitens* | NC_023349 |
|  | *Dermacentor* | *Dermacentor sp.* | OM368308 |
|  | *Dermacentor* | *Dermacentor (Indocentor) sp.* | OM368301 |
|  | *Dermacentor* | *Dermacentor rhinocerinus* | KY457527 |
|  | *Dermacentor* | *Dermacentor parumapertus* | PQ664580 |
|  | *Hyalomma* | *Hyalomma rufipes* | MW884229 |
|  | *Hyalomma* | *Hyalomma marginatum* | NC_056189 |
|  | *Hyalomma* | *Hyalomma asiaticum* | OR208589 |
|  | *Hyalomma* | *Hyalomma scupense* | OM368314 |
|  | *Hyalomma* | *Hyalomma excavatum* | MW546284 |
|  | *Hyalomma* | *Hyalomma anatolicum* | MW546283 |
|  | *Hyalomma* | *Hyalomma aegyptium* | MW546280 |
|  | *Hyalomma* | *Hyalomma truncatum* | KY457529 |
|  | *Haemaphysalis* | *Haemaphysalis taiwana* | OR778104 |
|  | *Haemaphysalis* | *Haemaphysalis flava* | AB075954 |
|  | *Haemaphysalis* | *Haemaphysalis hystricis* | NC_039765 |
|  | *Haemaphysalis* | *Haemaphysalis concinna* | NC_034785 |
|  | *Haemaphysalis* | *Haemaphysalis inermis* | NC_020335 |
|  | *Haemaphysalis* | *Haemaphysalis formosensis* | NC_020334 |
|  | *Haemaphysalis* | *Haemaphysalis tibetensis* | OM049539 |
|  | *Haemaphysalis* | *Haemaphysalis qinghaiensis* | OK094412 |
|  | *Haemaphysalis* | *Haemaphysalis bancrofti* | NC_041076 |
|  | *Haemaphysalis* | *Haemaphysalis warburtoni* | NC_084204 |
|  | *Haemaphysalis* | *Haemaphysalis colasbelcouri* | NC_062164 |
|  | *Haemaphysalis* | *Haemaphysalis nepalensis* | NC_064124 |
|  | *Haemaphysalis* | *Haemaphysalis danieli* | NC_062065 |
|  | *Haemaphysalis* | *Haemaphysalis mageshimaensis* | NC_062163 |
|  | *Haemaphysalis* | *Haemaphysalis cornigera* | NC_062162 |
|  | *Haemaphysalis* | *Haemaphysalis kitaokai* | NC_062161 |
|  | *Haemaphysalis* | *Haemaphysalis campanulata* | NC_062159 |
|  | *Haemaphysalis* | *Haemaphysalis doenitzi* | NC_062158 |
|  | *Haemaphysalis* | *Haemaphysalis sulcata* | NC_062063 |
|  | *Haemaphysalis* | *Haemaphysalis punctata* | NC_062064 |
|  | *Haemaphysalis* | *Haemaphysalis bispinosa* | OP383037 |
|  | *Haemaphysalis* | *Haemaphysalis montgomeryi* | NC_058312 |
|  | *Haemaphysalis* | *Haemaphysalis kolonini* | MZ054209 |
|  | *Haemaphysalis* | *Haemaphysalis japonica* | NC_037246 |
|  | *Haemaphysalis* | *Haemaphysalis yeni* | OL741745 |
|  | *Haemaphysalis* | *Haemaphysalis longicornis* | OL741744 |
|  | *Haemaphysalis* | *Haemaphysalis megaspinosa* | LC567953 |
|  | *Haemaphysalis* | *Haemaphysalis eleonorae* | PP059219 |
|  | *Haemaphysalis* | *Haemaphysalis novaeguineae* | NC_087880 |
|  | *Ixodes* | *Ixodes kohlsi* | NC_082161 |
|  | *Ixodes* | *Ixodes ornithorhynchi* | NC_082160 |
|  | *Ixodes* | *Ixodes anatis* | NC_082159 |
|  | *Ixodes* | *Ixodes loricatus* | NC_082158 |
|  | *Ixodes* | *Ixodes pacificus* | OR197647 |
|  | *Ixodes* | *Ixodes vespertilionis* | MW411447 |
|  | *Ixodes* | *Ixodes cornuatus* | NC_062630 |
|  | *Ixodes* | *Ixodes trichosuri* | NC_062633 |
|  | *Ixodes* | *Ixodes myrmecobii* | NC_062632 |
|  | *Ixodes* | *Ixodes hirsti* | NC_062631 |
|  | *Ixodes* | *Ixodes confusus* | NC_062629 |
|  | *Ixodes* | *Ixodes nipponensis* | NC_058242 |
|  | *Ixodes* | *Ixodes pavlovskyi* | NC_023831 |
|  | *Ixodes* | *Ixodes acutitarsus* | NC_061225 |
|  | *Ixodes* | *Ixodes ovatus* | OM317739 |
|  | *Ixodes* | *Ixodes (Pholeoixodes) sp.* | MW021452 |
|  | *Ixodes* | *Ixodes fecialis* | NC_062628 |
|  | *Ixodes* | *Ixodes woyliei* | NC_062627 |
|  | *Ixodes* | *Ixodes barkeri* | NC_062626 |
|  | *Ixodes* | *Ixodes australiensis* | NC_062625 |
|  | *Ixodes* | *Ixodes uriae* | NC_006078 |
|  | *Ixodes* | *Ixodes persulcatus* | NC_004370 |
|  | *Ixodes* | *Ixodes scapularis* | MZ645749 |
|  | *Ixodes* | *Ixodes ricinus* | NC_018369 |
|  | *Ixodes* | *Ixodes crenulatus* | OR872332 |
|  | *Ixodes* | *Ixodes columnae* | OL741746 |
|  | *Ixodes* | *Ixodes kuntzi* | NC_062157 |
|  | *Ixodes* | *Ixodes sinensis* | NC_062059 |
|  | *Ixodes* | *Ixodes nuttallianus* | NC_062062 |
|  | *Ixodes* | *Ixodes simplex* | NC_062060 |
|  | *Ixodes* | *Ixodes granulatus* | NC_061226 |
|  | *Ixodes* | *Ixodes angustus* | NC_067905 |
|  | *Ixodes* | *Ixodes rubicundus* | NC_067903 |
|  | *Ixodes* | *Ixodes tasmani* | NC_041086 |
|  | *Ixodes* | *Ixodes holocyclus* | NC_005293 |
|  | *Ixodes* | *Ixodes hexagonus* | NC_002010 |
|  | *Ixodes* | *Ixodes trianguliceps* | PP533213 |
|  | *Rhipicephalus* | *Rhipicephalus rutilus* | NC_072952 |
|  | *Rhipicephalus* | *Rhipicephalus sanguineus* | OQ184024 |
|  | *Rhipicephalus* | *Rhipicephalus turanicus* | OQ184023 |
|  | *Rhipicephalus* | *Rhipicephalus linnaei* | OM994391 |
|  | *Rhipicephalus* | *Rhipicephalus camicasi* | NC_061616 |
|  | *Rhipicephalus* | *Rhipicephalus appendiculatus* | NC_052829 |
|  | *Rhipicephalus* | *Rhipicephalus pumilio* | NC_084205 |
|  | *Rhipicephalus* | *Rhipicephalus haemaphysaloides* | NC_062072 |
|  | *Rhipicephalus* | *Rhipicephalus bursa* | OR773535 |
|  | *Rhipicephalus* | *Rhipicephalus zambeziensis* | NC_067930 |
|  | *Rhipicephalus* | *Rhipicephalus simus* | NC_067929 |
|  | *Rhipicephalus* | *Rhipicephalus maculatus* | NC_067928 |
|  | *Rhipicephalus* | *Rhipicephalus evertsi* | NC_067927 |
|  | *Rhipicephalus* | *Rhipicephalus microplus* | MK234703 |
|  | *Rhipicephalus* | *Rhipicephalus decoloratus* | NC_052828 |
|  | *Rhipicephalus* | *Rhipicephalus annulatus* | NC_067926 |
|  | *Rhipicephalus* | *Rhipicephalus geigyi* | NC_023350 |
|  | *Rhipicephalus* | *Rhipicephalus australis* | NC_023348 |
|  | *Rhipicephalus* | *Rhipicephalus secundus* | PP970533 |
|  | *Rhipicentor* | *Rhipicentor nuttalli* | NC_039828 |
| Argasidae | *Argas* | *Argas africolumbae* | NC_019642 |
|  | *Ornithodoros* | *Ornithodoros compactus* | NC_067908 |
| Nuttalliellidae | *Nuttalliella* | *Nuttalliella namaqua* | NC_019663 |
| Limulidae | *Limulus* | *Limulus polyphemus* | NC_003057 |
