# Supplementary material for: Comparative mitogenomic and phylogenetic insights from four newly sequenced tick mitochondrial genomes
Source: Front Vet Sci. 2026 Jan 22;12:1678349. doi: 10.3389/fvets.2025.1678349 (PMC12872541; doi:10.3389/fvets.2025.1678349)
Supplement: Supplementary file 5 [file Table_5.docx]

| Species | Length (bp) | A+T (%) | AT skew | GC skew |
| --- | --- | --- | --- | --- |
| *Amblyomma cajennense* | 14, 661 | 75.96 | -0.03 | -0.16 |
| *Amblyomma parvum* | 14, 810 | 77.00 | -0.02 | -0.18 |
| *Amblyomma neumanni* | 14, 803 | 77.02 | -0.02 | -0.17 |
| *Amblyomma parvitarsum* | 14, 655 | 79.06 | -0.04 | -0.15 |
| *Amblyomma tigrinum* | 14, 681 | 79.44 | -0.03 | -0.14 |
| *Amblyomma dubitatum* | 14, 658 | 78.40 | -0.02 | -0.16 |
| *Amblyomma naponense* | 14, 709 | 78.96 | -0.02 | -0.17 |
| *Amblyomma boeroi* | 14, 760 | 75.00 | -0.03 | -0.12 |
| *Amblyomma argentinae* | 14, 760 | 77.61 | -0.03 | -0.13 |
| *Amblyomma dissimile* | 14, 780 | 80.50 | -0.03 | -0.13 |
| *Amblyomma patinoi* | 14, 780 | 76.48 | -0.03 | -0.16 |
| *Amblyomma tonelliae* | 14, 780 | 77.37 | -0.01 | -0.17 |
| *Amblyomma ovale* | 14, 709 | 76.38 | -0.03 | -0.13 |
| *Amblyomma javanense* | 14, 740 | 79.49 | -0.01 | -0.15 |
| *Amblyomma sculptum* | 14, 611 | 76.10 | -0.03 | -0.16 |
| *Amblyomma americanum* | 14, 865 | 76.78 | -0.03 | -0.15 |
| *Amblyomma triguttatum* | 14, 798 | 78.40 | -0.02 | -0.13 |
| *Amblyomma mixtum* | 14, 808 | 76.41 | -0.03 | -0.16 |
| *Amblyomma maculatum* | 14, 750 | 78.75 | -0.04 | -0.14 |
| *Amblyomma testudinarium* | 14, 808 | 78.49 | -0.01 | -0.16 |
| *Amblyomma geoemydae* | 14, 678 | 77.46 | -0.03 | -0.15 |
| *Amblyomma nuttalli* | 14, 716 | 79.08 | -0.03 | -0.18 |
| *Amblyomma latum* | 14, 727 | 79.84 | -0.01 | -0.15 |
| *Amblyomma gervaisi* | 14, 779 | 79.16 | -0.02 | -0.17 |
| *Amblyomma tholloni* | 14, 731 | 75.46 | -0.02 | -0.15 |
| *Amblyomma hebraeum* | 14, 780 | 77.26 | -0.02 | -0.16 |
| *Amblyomma sparsum* | 14, 681 | 79.08 | -0.03 | -0.17 |
| *Amblyomma sp.* | 14, 760 | 78.54 | -0.01 | -0.17 |
| *Amblyomma marmoreum* | 14, 667 | 77.75 | -0.03 | -0.16 |
| *Amblyomma limbatum* | 14, 823 | 80.35 | -0.02 | -0.15 |
| *Amblyomma albolimbatum* | 14, 835 | 80.10 | -0.02 | -0.15 |
| *Amblyomma variegatum* | 14, 636 | 76.88 | -0.02 | -0.15 |
| *Amblyomma triste* | 14, 808 | 78.97 | -0.03 | -0.14 |
| *Amblyomma papuanum* | 14, 757 | 76.40 | -0.02 | -0.15 |
| *Amblyomma postoculatum* | 14, 749 | 76.47 | -0.03 | -0.12 |
| *Amblyomma nitidum* | 14, 757 | 77.17 | -0.03 | -0.13 |
| *Amblyomma calabyi* | 14, 818 | 80.19 | -0.02 | -0.15 |
| *Amblyomma breviscutatum* | 14, 762 | 74.18 | -0.02 | -0.15 |
| *Aponomma fimbriatum* | 14, 705 | 77.67 | -0.02 | -0.12 |
| *Archaeocroton sphenodonti* | 14, 772 | 77.78 | -0.03 | -0.12 |
| *Bothriocroton undatum* | 14, 769 | 76.90 | -0.04 | -0.10 |
| *Bothriocroton concolor* | 14, 809 | 75.14 | -0.02 | -0.13 |
| *Robertsicus elaphensis* | 14, 627 | 80.45 | -0.03 | -0.14 |
| *Dermacentor albipictus* | 14, 822 | 77.54 | -0.02 | -0.15 |
| *Dermacentor reticulatus* | 14, 811 | 78.30 | -0.01 | -0.15 |
| *Dermacentor variabilis* | 14, 837 | 78.57 | -0.03 | -0.15 |
| *Dermacentor andersoni* | 14, 806 | 78.91 | -0.02 | -0.18 |
| *Dermacentor auratus* | 14, 766 | 77.16 | -0.02 | -0.14 |
| *Dermacentor everestianus* | 15, 191 | 78.80 | -0.03 | -0.11 |
| *Dermacentor nuttalli* | 15, 086 | 78.93 | -0.01 | -0.17 |
| *Dermacentor silvarum* | 14, 945 | 78.78 | -0.01 | -0.16 |
| *Dermacentor sinicus* | 14, 996 | 79.03 | -0.02 | -0.16 |
| *Dermacentor niveus* | 15, 110 | 78.54 | -0.02 | -0.15 |
| *Dermacentor steini* | 14, 773 | 76.86 | -0.02 | -0.13 |
| *Dermacentor marginatus* | 15, 178 | 78.40 | -0.02 | -0.16 |
| *Dermacentor nitens* | 14, 839 | 77.42 | -0.02 | -0.16 |
| *Dermacentor sp.* | 15, 307 | 78.32 | -0.02 | -0.16 |
| *Dermacentor (Indocentor) sp.* | 14, 796 | 77.64 | -0.01 | -0.15 |
| *Dermacentor rhinocerinus* | 14, 708 | 76.75 | -0.03 | -0.14 |
| *Dermacentor parumapertus* | 14, 848 | 79.14 | 0.02 | 0.18 |
| *Hyalomma rufipes* | 14, 761 | 79.70 | -0.03 | -0.12 |
| *Hyalomma marginatum* | 14, 764 | 79.76 | -0.03 | -0.12 |
| *Hyalomma asiaticum* | 14, 722 | 78.21 | -0.03 | -0.14 |
| *Hyalomma scupense* | 14, 721 | 79.80 | -0.03 | -0.13 |
| *Hyalomma excavatum* | 14, 733 | 78.12 | -0.02 | -0.14 |
| *Hyalomma anatolicum* | 14, 731 | 78.07 | -0.02 | -0.14 |
| *Hyalomma aegyptium* | 14, 707 | 79.13 | -0.02 | -0.16 |
| *Hyalomma truncatum* | 14, 731 | 77.20 | -0.02 | -0.14 |
| *Haemaphysalis taiwana* | 14, 685 | 72.32 | 0.02 | -0.36 |
| *Haemaphysalis flava* | 14, 686 | 76.91 | -0.02 | -0.12 |
| *Haemaphysalis hystricis* | 14, 716 | 77.22 | -0.01 | -0.14 |
| *Haemaphysalis concinna* | 14, 675 | 77.98 | -0.01 | -0.16 |
| *Haemaphysalis inermis* | 14, 846 | 78.82 | -0.01 | -0.15 |
| *Haemaphysalis formosensis* | 14, 676 | 78.29 | -0.01 | -0.14 |
| *Haemaphysalis tibetensis* | 14, 714 | 77.70 | -0.01 | -0.14 |
| *Haemaphysalis qinghaiensis* | 14, 533 | 77.65 | -0.02 | -0.13 |
| *Haemaphysalis bancrofti* | 14, 673 | 78.35 | -0.01 | -0.13 |
| *Haemaphysalis warburtoni* | 14, 695 | 77.83 | -0.02 | -0.14 |
| *Haemaphysalis colasbelcouri* | 14, 885 | 77.95 | -0.01 | -0.16 |
| *Haemaphysalis nepalensis* | 14, 720 | 77.75 | -0.01 | -0.15 |
| *Haemaphysalis danieli* | 14, 739 | 81.06 | -0.01 | -0.13 |
| *Haemaphysalis mageshimaensis* | 14, 721 | 78.07 | -0.01 | -0.14 |
| *Haemaphysalis cornigera* | 14, 681 | 78.08 | -0.01 | -0.13 |
| *Haemaphysalis kitaokai* | 14, 936 | 77.32 | 0.00 | -0.16 |
| *Haemaphysalis campanulata* | 14, 691 | 78.16 | 0.00 | -0.13 |
| *Haemaphysalis doenitzi* | 14, 671 | 77.42 | -0.01 | -0.15 |
| *Haemaphysalis sulcata* | 14, 679 | 76.40 | -0.01 | -0.16 |
| *Haemaphysalis punctata* | 14, 697 | 78.01 | -0.01 | -0.16 |
| *Haemaphysalis bispinosa* | 14, 732 | 78.74 | -0.01 | -0.13 |
| *Haemaphysalis montgomeryi* | 14, 681 | 78.46 | 0.00 | -0.17 |
| *Haemaphysalis kolonini* | 14, 948 | 78.46 | -0.01 | -0.14 |
| *Haemaphysalis japonica* | 14, 685 | 77.58 | -0.02 | -0.12 |
| *Haemaphysalis yeni* | 14, 714 | 77.57 | 0.00 | -0.14 |
| *Haemaphysalis longicornis* | 14, 696 | 77.20 | -0.01 | -0.14 |
| *Haemaphysalis megaspinosa* | 14, 680 | 77.68 | -0.02 | -0.12 |
| *Haemaphysalis eleonorae* | 14, 688 | 79.21 | -0.02 | -0.12 |
| *Haemaphysalis novaeguineae* | 14, 681 | 78.04 | -0.02 | -0.12 |
| *Ixodes kohlsi* | 14, 578 | 77.38 | -0.01 | -0.23 |
| *Ixodes ornithorhynchi* | 15, 216 | 76.39 | 0.02 | -0.32 |
| *Ixodes anatis* | 15, 164 | 76.98 | 0.01 | -0.31 |
| *Ixodes loricatus* | 14, 463 | 74.12 | -0.01 | -0.31 |
| *Ixodes pacificus* | 14, 578 | 76.89 | -0.03 | -0.26 |
| *Ixodes vespertilionis* | 14, 559 | 74.94 | 0.02 | -0.31 |
| *Ixodes cornuatus* | 14, 985 | 77.81 | -0.01 | -0.27 |
| *Ixodes trichosuri* | 15, 001 | 78.30 | -0.01 | -0.28 |
| *Ixodes myrmecobii* | 14, 995 | 78.24 | -0.01 | -0.26 |
| *Ixodes hirsti* | 15, 040 | 77.35 | -0.01 | -0.30 |
| *Ixodes confusus* | 14, 939 | 77.83 | -0.01 | -0.28 |
| *Ixodes nipponensis* | 14, 505 | 77.64 | -0.03 | -0.27 |
| *Ixodes pavlovskyi* | 14, 575 | 78.09 | -0.03 | -0.26 |
| *Ixodes acutitarsus* | 14, 475 | 78.31 | -0.02 | -0.29 |
| *Ixodes ovatus* | 14, 520 | 74.93 | 0.00 | -0.30 |
| *Ixodes (Pholeoixodes) sp.* | 14, 543 | 72.28 | 0.02 | -0.36 |
| *Ixodes fecialis* | 15, 256 | 77.5 | -0.01 | -0.30 |
| *Ixodes woyliei* | 15, 062 | 77.78 | 0.01 | -0.29 |
| *Ixodes barkeri* | 15, 259 | 77.83 | 0.01 | -0.28 |
| *Ixodes australiensis* | 15, 217 | 78.54 | 0.00 | -0.25 |
| *Ixodes uriae* | 15, 053 | 74.79 | 0.01 | -0.11 |
| *Ixodes persulcatus* | 14, 539 | 77.34 | -0.02 | -0.27 |
| *Ixodes scapularis* | 14, 537 | 78.19 | -0.03 | -0.24 |
| *Ixodes ricinus* | 14, 566 | 78.66 | -0.02 | -0.26 |
| *Ixodes crenulatus* | 15, 548 | 76.87 | -0.01 | -0.30 |
| *Ixodes columnae* | 14, 524 | 78.32 | -0.02 | -0.26 |
| *Ixodes kuntzi* | 14, 524 | 78.28 | -0.16 | -0.26 |
| *Ixodes sinensis* | 14, 534 | 77.58 | -0.03 | -0.27 |
| *Ixodes nuttallianus* | 14, 604 | 74.63 | 0.02 | -0.29 |
| *Ixodes simplex* | 14, 556 | 75.87 | 0.01 | -0.31 |
| *Ixodes granulatus* | 14, 540 | 77.95 | 0.02 | -0.27 |
| *Ixodes angustus* | 14, 529 | 74.53 | 0.03 | -0.34 |
| *Ixodes rubicundus* | 14, 532 | 79.89 | -0.03 | -0.20 |
| *Ixodes tasmani* | 15, 227 | 77.92 | 0.00 | -0.29 |
| *Ixodes holocyclus* | 15, 007 | 77.38 | -0.01 | -0.25 |
| *Ixodes hexagonus* | 14, 539 | 72.66 | 0.03 | -0.37 |
| *Ixodes trianguliceps* | 14, 558 | 78.40 | 0.00 | -0.26 |
| *Rhipicephalus rutilus* | 14, 718 | 78.06 | -0.03 | -0.11 |
| *Rhipicephalus sanguineus* | 14, 719 | 78.11 | -0.03 | -0.01 |
| *Rhipicephalus turanicus* | 14, 720 | 77.81 | -0.03 | -0.11 |
| *Rhipicephalus linnaei* | 14, 711 | 77.44 | -0.02 | -0.12 |
| *Rhipicephalus camicasi* | 14, 725 | 77.56 | -0.02 | -0.10 |
| *Rhipicephalus appendiculatus* | 15, 001 | 77.81 | -0.02 | -0.11 |
| *Rhipicephalus pumilio* | 14, 751 | 77.31 | -0.02 | -0.12 |
| *Rhipicephalus haemaphysaloides* | 14, 744 | 77.66 | -0.03 | -0.10 |
| *Rhipicephalus bursa* | 14, 741 | 77.61 | -0.02 | -0.12 |
| *Rhipicephalus zambeziensis* | 14, 739 | 77.26 | -0.02 | -0.11 |
| *Rhipicephalus simus* | 14, 721 | 76.20 | -0.03 | -0.11 |
| *Rhipicephalus maculatus* | 14, 714 | 74.57 | -0.03 | -0.12 |
| *Rhipicephalus evertsi* | 14, 743 | 76.42 | -0.03 | -0.12 |
| *Rhipicephalus microplus* | 14, 903 | 79.47 | -0.02 | -0.12 |
| *Rhipicephalus decoloratus* | 15, 268 | 79.40 | -0.03 | -0.11 |
| *Rhipicephalus annulatus* | 15, 156 | 80.26 | -0.02 | -0.01 |
| *Rhipicephalus geigyi* | 14, 948 | 80.37 | -0.02 | -0.12 |
| *Rhipicephalus australis* | 14, 891 | 79.89 | -0.03 | -0.10 |
| *Rhipicephalus secundus* | 14, 714 | 77.01 | -0.03 | -0.11 |
| *Rhipicentor nuttalli* | 14,779 | 78.27 | -0.04 | -0.14 |
