# Supplementary figures and images for: Comparative mitogenomic and phylogenetic insights from four newly sequenced tick mitochondrial genomes
Source: Front Vet Sci. 2026 Jan 22;12:1678349. doi: 10.3389/fvets.2025.1678349 (PMC12872541; doi:10.3389/fvets.2025.1678349)

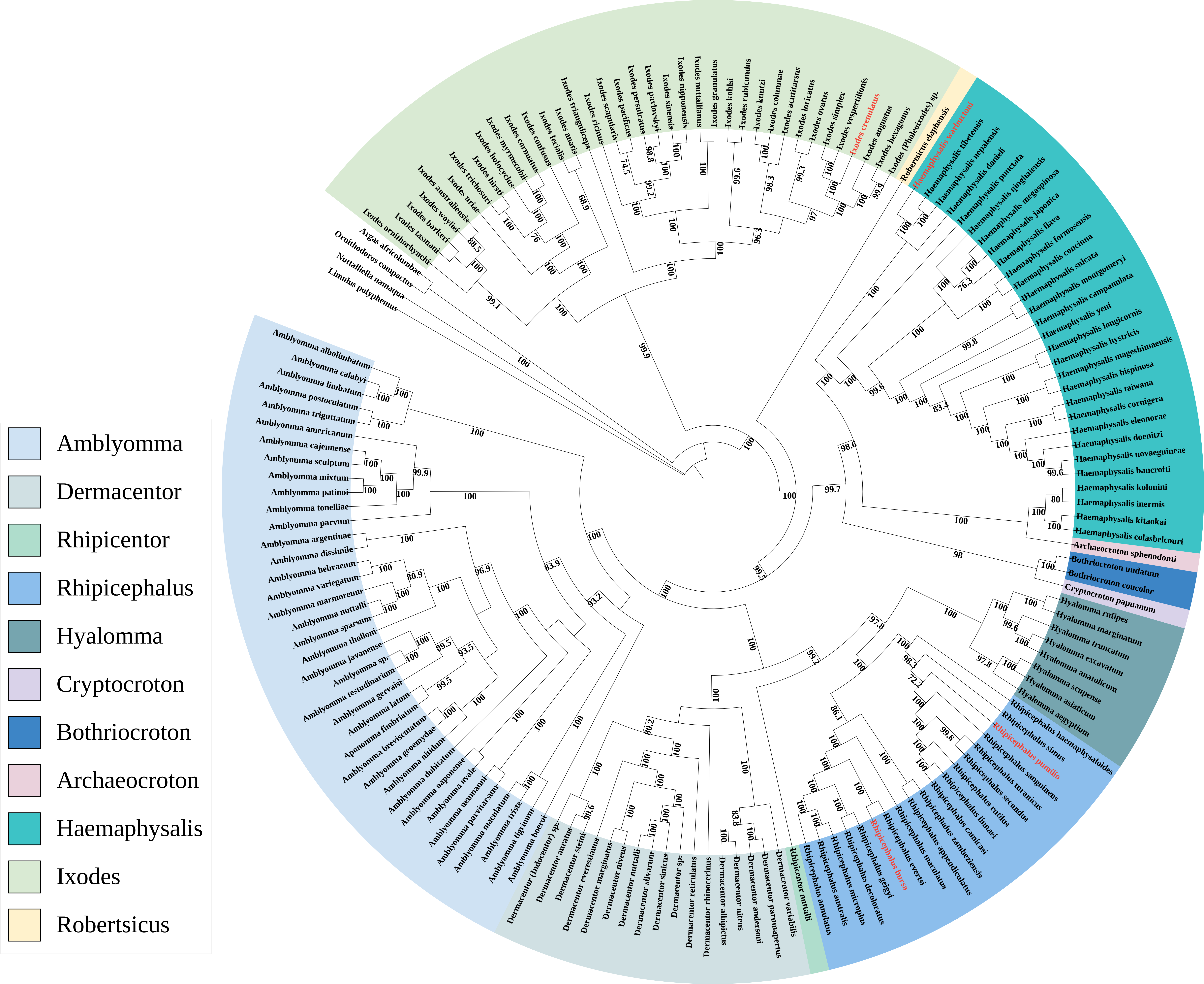

Supplement: Supplementary file 6 [file Image_1.png]
